# TWIRLS, a knowledge‐mining technology, suggests a possible mechanism for the pathological changes in the human host after coronavirus infection via ACE2

**DOI:** 10.1002/ddr.21717

**Published:** 2020-07-13

**Authors:** Xiaoyang Ji, Wenting Tan, Chunming Zhang, Yubo Zhai, Yiching Hsueh, Zhonghai Zhang, Chunli Zhang, Yanqiu Lu, Bo Duan, Guangming Tan, Renhua Na, Guohong Deng, Gang Niu

**Affiliations:** ^1^ Key Laboratory of Animal Genetics, Breeding and Reproduction of Inner Mongolia Autonomous Region College of Animal Science Inner Mongolia Agricultural University Hohhot China; ^2^ Joint Turing‐Darwin Laboratory of Phil Rivers Technology Ltd. and Institute of Computing Technology Chinese Academy of Sciences Beijing China; ^3^ Department of Computational Biology, Phil Rivers Technology Ltd Beijing China; ^4^ Department of Infectious Diseases Southwest Hospital, Third Military Medical University (Army Medical University) Chongqing China; ^5^ Institute of Computing Technology Chinese Academy of Sciences Beijing China; ^6^ West Institute of Computing Technology Chinese Academy of Sciences Chongqing China; ^7^ Department of Infectious Diseases Chongqing Public Health Medical Center Chongqing China; ^8^ University of Chinese Academy of Sciences Beijing China

**Keywords:** coronavirus, cytokine storm, literature mining, renin‐angiotensin system, topic inference

## Abstract

Faced with the current large‐scale public health emergency, collecting, sorting, and analyzing biomedical information related to the “SARS‐CoV‐2” should be done as quickly as possible to gain a global perspective, which is a basic requirement for strengthening epidemic control capacity. However, for human researchers studying viruses and hosts, the vast amount of information available cannot be processed effectively and in a timely manner, particularly if our scientific understanding is also limited, which further lowers the information processing efficiency. We present TWIRLS (Topic‐wise inference engine of massive biomedical literatures), a method that can deal with various scientific problems, such as liver cancer, acute myeloid leukemia, and so forth, which can automatically acquire, organize, and classify information. Additionally, this information can be combined with independent functional data sources to build an inference system via a machine‐based approach, which can provide relevant knowledge to help human researchers quickly establish subject cognition and to make more effective decisions. Using TWIRLS, we automatically analyzed more than three million words in more than 14,000 literature articles in only 4 hr. We found that an important regulatory factor angiotensin‐converting enzyme 2 (ACE2) may be involved in host pathological changes on binding to the coronavirus after infection. On triggering functional changes in ACE2/AT2R, the cytokine homeostasis regulation axis becomes imbalanced via the Renin‐Angiotensin System and IP‐10, leading to a cytokine storm. Through a preliminary analysis of blood indices of COVID‐19 patients with a history of hypertension, we found that non‐ARB (Angiotensin II receptor blockers) users had more symptoms of severe illness than ARB users. This suggests ARBs could potentially be used to treat acute lung injury caused by coronavirus infection.

## 1 INTRODUCTION

The sudden outbreak of a new coronavirus (SARS‐CoV‐2) at the end of December 2019 is currently posing huge health challenges worldwide. The SARS‐CoV‐2 virus causes severe respiratory disease (COVID‐19) that can quickly spread from person to person and in some cases lead to death. Researchers have found that both the SARS‐CoV‐2 and SARS coronaviruses invade human cells in target tissues in a similar manner via high‐affinity binding to angiotensin‐converting enzyme 2 (ACE2) (Zhou et al., 2020). In recent epidemiological investigations of the spread of SARS‐CoV‐2 and a preliminary study of the clinical characteristics of this disease (Chan et al., 2020; Chen et al., 2020; Pan et al., 2020; Wei et al., 2020; Zhu et al., 2020), researchers have found that patients infected with the new coronavirus have severe symptoms similar to that of the SARS infection. The first clinical case reports of SARS‐CoV‐2 infections in China revealed “cytokine storms” in critically ill patients (Huang et al., 2020; Wan et al., 2020). However, the mechanism of the viral infection and pathological changes in the immune system are still not known. The sooner this information is added to the current clinical knowledge on these viruses, the better the control and treatment of this disease.

Here, we present an automated topic‐wise inference method called TWIRLS (Topic‐wise inference engine of massive biomedical literatures), to help human researchers to quickly establish topic‐cognition of interest and solve different scientific problems. In this study, we constructed the “coronavirus” knowledge graph using the TWIRLS system. First, TWIRLS can process and summarize the massive biomedical literature on coronaviruses, and then collect, classify, and analyze reported coronavirus studies to reveal host‐related entities based on the distribution of specific genes in the text of the articles. By combining with general protein interaction data, links between certain functional cellular/physiological components can be inferred to fill in the knowledge gaps on the probable mechanisms of host pathological changes. By analyzing the coronavirus literature, TWIRLS was able to reveal that the binding of the coronavirus spike proteins to ACE2 would cause an imbalance in the Renin‐Angiotensin System (RAS). When the level of Ang II is elevated, the angiotensin‐stimulated AT1R leads to increased pulmonary vascular permeability, which triggers cytokine storm and then eventually results acute lung injury in the host (Imai et al., 2005; Kuba et al., 2005). Therefore, TWIRLS can guide human researchers by providing further potential therapeutic target information based on the regulation of RAS for the treatment of acute viral lung injury.

## 2 METHODS

### 2.1 Construction of the data interface

**FIGURE 1:**
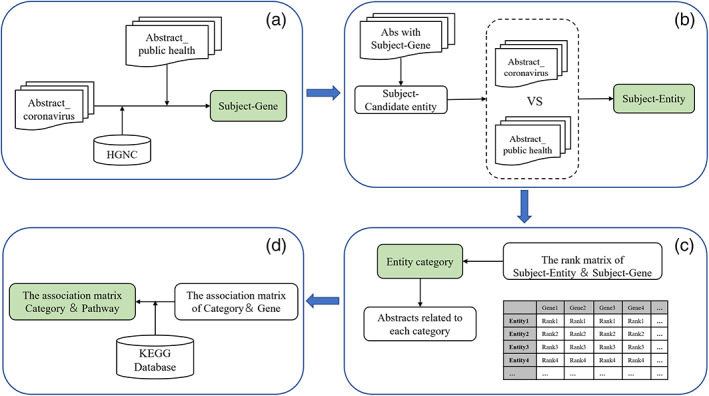
Flow chart of the knowledge‐driven literature mining method, the basic steps of the literature mining includes: (a) identify genes with accurate relevance to the subject, (b) identify entities with accurate relevance to the subject, (c) entities were classified by calculating the association strength between genes and entities, (d) alignment with KEGG database to establish an association matrix between pathways and entity‐categories

We used PubMed, the most widely used biological literature database, as the resource for text mining. The schematic representation of the overall study design is shown in Figure 1 and can be summarized in the following steps.

### 2.2 Corpus and dictionary organization

The dataset used in this pipeline was derived from the text of articles from PubMed. First, PubMed was searched for articles containing the subject keyword “coronavirus” including titles, abstracts, and author and affiliation information. The search results were downloaded in txt format for compiling into structured information. The text in the subject abstract set was organized and cleaned, and assigned to specific corpuses related to the coronavirus (specific corpus), and then compiled into the subject dictionary. To enhance the accuracy of effective entities associated with the key word, we also constructed the control group which is a random corpus with “public health” as the key word. For balancing the amount of information, we randomly selected the same amount of text as the subject abstract set from the control group before statistical analysis.

### 2.3 Identification of genes precisely related to the subject “coronavirus”

Biological entity identification is a key step in the literature mining process. To validate the functionality of the extracted entities, we first compared the entities from the subject dictionary with the human official gene symbols in the Hugo Gene Nomenclature Commission (HGNC) database to generate subject candidate genes using standard nomenclature. In addition, the entities in the abstract were capitalized to avoid errors in the identification process. To obtain widely used gene entities that are precisely related to the subject and to determine the significance of the gene distribution in the specific texts, we calculated the difference in the distribution proportions. We first searched for the subject candidate genes in the subject dictionary and in the randomized control dictionary, respectively. We then counted the number of abstracts containing each subject candidate gene in each abstract set, respectively. Finally, we calculated the odds ratio of each subject candidate gene and sorted them into a list of precisely related genes referred to as coronavirus study‐specific host genes (CSHG).

### 2.4 Identification of all entities correctly related to the subject “coronavirus”

Similar to the process of identifying CSHG, we calculated whether entities were significantly distributed in a specific corpus as the coronavirus study‐specific entities (CSSE). We counted the number of texts containing each CSHG in a specific corpus, and then counted the number of each candidate entity in the corpus subset. Next, we randomly selected the same amount of text from the random control corpus and then counted the number of each candidate entity in this subset of the random corpus. This was repeated 100–10,000 times in the random corpus to generate candidate entities in the specified amount of text from the random distribution model. According to the central limit theorem, the distribution of random sampling averages of randomly distributed data always conforms to a normal distribution. Therefore, we can use the Z score to evaluate whether an entity is significant in a specific text. Here, we used a cutoff Z score > 6.

In addition, some entities mentioned in the abstracts are in singular or plural noun forms, or synonyms with multiple forms. Therefore, we automatically combined nouns with plural forms and homologous words with adjectives and adverb roots into the same subject‐related entities and assigned them the same number. For example, synonymous entities such as coronaviral, coronavirus, coronaviruses were grouped into one entity called coronavirus and assigned with one number (see entity number in Table S1, Sheet 1 first column). A previous method of merging synonymous entities based on a dictionary (Cook & Jensen, 2019; Hettne et al., 2010) relied on the integrity of that dictionary, and also required a long retrieval time. To automatically solve the synonymous entity problem, TWIRLS classifies similar strings based on whether there is a significant statistical association between the character blocks in a set of candidate entities including various synonymous entities.

### 2.5 Programming language and efficiency

Part of the algorithm was developed using the MatLab programming environment and Python language. Algorithm efficiency improvements and the targeted parallel acceleration module were developed in C/C++ language. In our analysis, the automated text analysis took about 4 hr to complete on a workstation with an Intel Xeon CPU E5‐2690 v4 X2 (28 cores) and 128 GB of memory.

### 2.6 Clinical data collection

In this study, we collected the medical records of 92 patients with COVID‐19:90 patients with COVID‐19 admitted to the Chongqing Public Health Medical Center (Chongqing, China) from Jan 24 to Mar 15, 2020 and two patients with COVID‐19 admitted to the Chongqing Southwest Hospital (Chongqing, China) from Jan 25 to Feb 28, 2020, all patients were diagnosed with COVID‐19‐related pneumonia based on the New Coronavirus Pneumonia Prevention guidelines (World Health Organization, 2020). This study was performed in accordance with guidelines approved by the Ethics Committees from the Institute of Basic Medical Sciences, Chinese Academy of Medical Sciences (002–2020). Demographic, clinical, treatment, and laboratory data were extracted from medical records. Patients with COVID‐19 were classified into mild, severe, and critical according to their condition based on the partial pressure of oxygen test. All data were independently checked by more than one physician.

## 3 RESULTS

### 3.1 Coronavirus specific entities and host genes

As of February 21, 2020, the PubMed database included 14,878 biomedical articles on coronaviruses. We obtained text data (referred to as the local samples) from all related peer reviewed articles published by human researchers that contained the keyword “coronavirus” including the title, abstracts, and author and affiliation information (total 3,182,687 words). The goal of the literature mining was to identify host genes and entities that are relevant to coronavirus research and to establish connections between them. An entity can refer to a word or phrase of the concept name including related concepts (e.g., virus structure and chemical composition, source of infection, and virus type). The gene names were defined using the mammalian official gene symbols in the Hugo Gene Naming Committee (HGNC) database. We directly retrieved 667 candidate genes from the local samples. By establishing a random distribution of one of the candidate genes in a control sample, the significance of this gene appearing in the local samples can be determined if the frequency of the current gene is an outlier of the random distribution of a control sample (see Methods for details). By calculating the odds ratio, we can also further determine the specificity of the association between this gene and the local samples. In this paper, we selected an odds ratio > 6 as the threshold for this judgment, which resulted in 123 coronavirus study‐specific host genes (CSHGs).

To determine the specificity of the entity, we made several choices in the different texts in the local samples. We removed numbers, symbols, verbs, and garbled characters to obtain clean versions of the local samples. The CSSE were then identified in only the clean texts containing CSHGs. Based on the clean selected samples, we next built a local dictionary of candidate CSSEs, which contained 49,293 words after deduplication. Before calculating the random distribution of each entity, we included the synonymous entities into a same entity number (including singular or plural words, active and passive forms, different tenses, suffixes that do not change the meaning, etc.).

**FIGURE 2:**
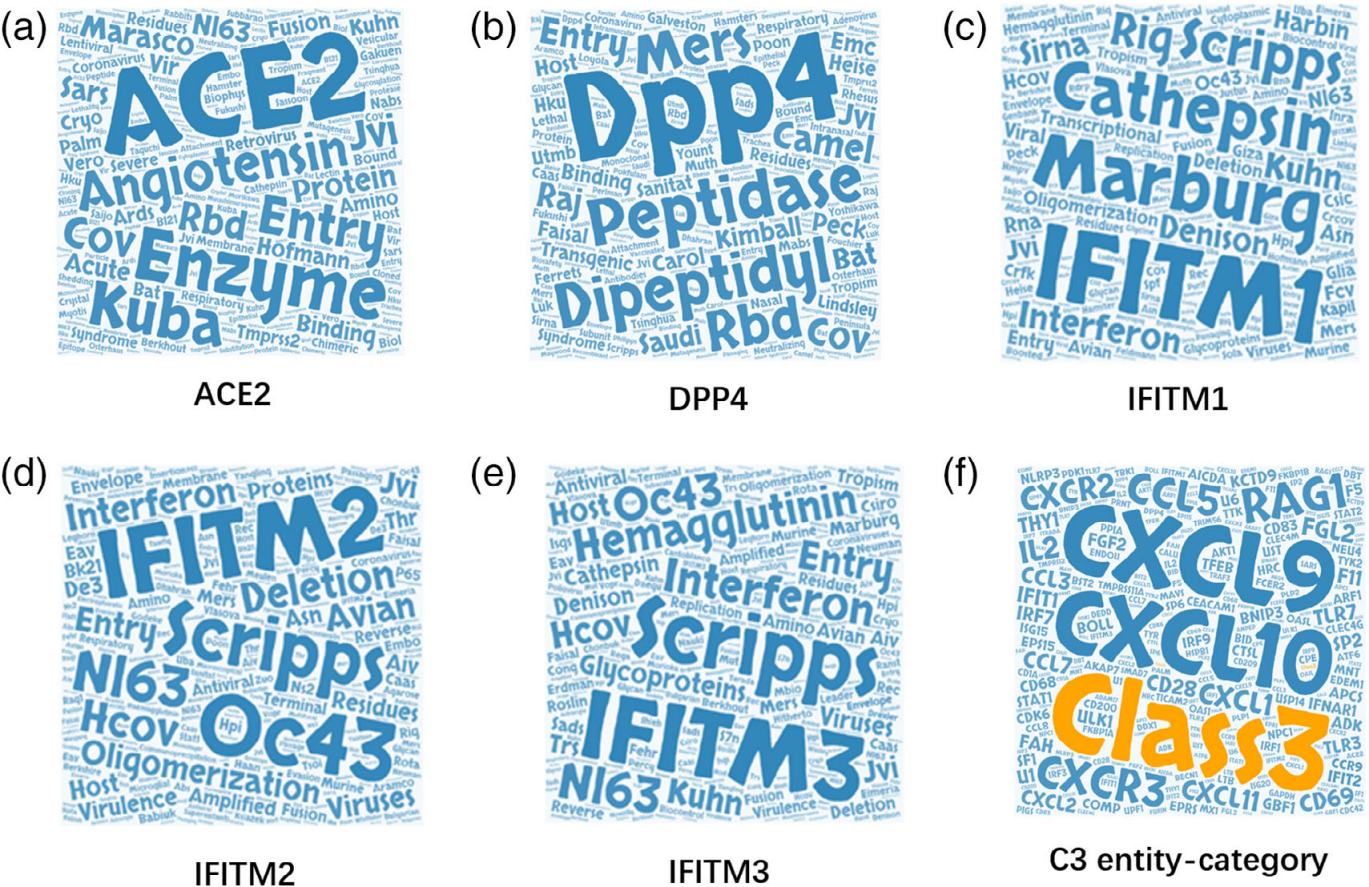
(a, b) The entity cloud (CSSE cloud) associated with ACE2 and DPP4 in the coronavirus knowledge graph. (c–e) The entity clouds of the three IFITMs family proteins (IFITM1‐3) in the coronavirus knowledge graph. (f) The gene cloud associated with coronavirus‐C3 entity category

After cleaning and processing, CSSEs were identified by TWIRLS using a similar method as described above for CSHG. For the candidate CSSE dictionary, a random distribution model for each entity was built by TWIRLS using the control samples. We identified 623 CSSEs (Table S1) based on the outliers discriminated by the random model and the calculated odds ratios. For example, TWIRLS found 100 CSSEs close to ACE2, the receptor of SARS and SARS‐CoV‐2 viruses (Figure 2a). The size of the entity represented the relative distance to ACE2, with a larger size indicating a closer distance to ACE2. Additionally, we also constructed the CSSE cloud of the human receptor gene DPP4 of the MERS virus (Figure 2b).

### 3.2 Entity categories and their labels: Human conclusions and enriched pathways

Although TWIRLS only identified 623 CSSEs after collation, the information is scattered in words, which limits the reconstruction of understandable mechanistic models. Accordingly, TWIRLS clusters CSSEs based on the rules defined by the CSHG distribution, as genetic level research can accurately answer and solve physiological and pathological problems. TWIRLS first calculates the specific co‐distribution between CSHGs in local samples, then determines the distance between each pair of CSSEs and performs dichotomy clustering according to the linkage relationship between CSSEs and CSHGs. This step classified the 623 entities into 32 categories represented as C0‐C31 (see category number in Table S1, Sheet 1 second column). In addition, for each category, TWIRLS also cited the top 10 most relevant references for human researchers (Table S2). Therefore, in any category, according to the CSSE and the most relevant literature, we can quickly provide “Labels of conclusion‐drawn‐by‐human‐researcher” (HR Labels) for this category. This label outlines the most relevant research directions of the current entity category. For example, for category C3, the HR label is “Neurotrophic Coronavirus Related to Immune‐Mediated Demyelination”. We have summarized the HR labels for the 32 entity categories in Table 1.

**TABLE 1:** Coronavirus‐entity category labels and genes associated with each category. MISC indicates the label cannot be summarized

Category	HR label
C0	MISC
C1	Canine coronavirus
C2	Porcine epidemic diarrhea (PED)
C3	Neurotropic coronavirus correlated with immune‐mediated demyelination
C4	Coronavirus that infects humans
C5	Coronavirus spike protein
C6	Protease enhances SARS‐CoV infection
C7	Monoclonal antibody to the coronavirus nucleocapsid protein
C8	SARS‐CoV genome
C9	Avian infectious bronchitis coronavirus
C10	Coronavirus and interferon
C11	Feline infectious peritonitis (FIP)
C12	Vectors of novel coronaviruses
C13	Mouse hepatitis virus
C14	Interaction between coronaviruses and receptors
C15	Coronavirus‐related vaccines
C16	Identification of MHC class I restricted T‐cell epitopes
C17	Transmissible gastroenteritis coronavirus
C18	SARS coronavirus inhibitors and diagnostic methods
C19	Coronavirus fusion with host cells and virus replication
C20	Gene therapy‐inhibition of coronavirus by antisense RNA, sense RNA and protein
C21	Imaging
C22	Cytotoxic T‐lymphocyte escape
C23	SARS coronavirus compound inhibitors
C24	Coronavirus studies using biophysical methods
C25	Detection of viral pathogenicity and distribution (RT‐PCR, immunohistochemistry and in situ hybridization)
C26	Coronavirus immunization
C27	Gastroenteritis virus and coronavirus
C28	Effects of coronavirus infection on the body
C29	Coronavirus detection evidence and methods
C30	Human respiratory coronavirus NL63
C31	The antibodies against SARS‐CoV

The relative position of any CSHG to a certain CSSE can be estimated by TWIRLS (see Table S1). As each category contains different entities, we can determine whether a certain CSHG is significantly closer to each entity in the current category based on the ranking matrix between CSHG and CSSE. For example, the average distance between ACE2 and each of the 92 entities in category C5 was first calculated and a random distribution model of the average distances between ACE2 and any of the 92 entities (3,000–5,000 times) was built. The average distances between ACE2 and entities in category C5 were then analyzed to determine those significantly less than or those deviating from the mean of the random distribution (Z score = −5.8416). The significance of each category associated with each CSHG was then determined by TWIRLS using a score ranging between −10 and + 10, with a smaller score indicating the current CSHG is more relevant to the current category (see the Z score matrix in Table S3). For an entity category, the associated CSHGs (e.g., ^Ci^CSHGs, where *i* represents the category number) can thus be selected using a Z score < −3. The Z scores describing the association between CSHG and any category is summarized in Table S3 and the category labels of all CSHGs are provided in Table S4.

Specifically, Spike proteins (S proteins) of different coronaviruses recognize different receptor molecules on human cells: ACE2 binds to S proteins in SARS and SARS‐CoV‐2 viruses, and DPP4 binds to S proteins in the MERS virus, FURIN restriction site on the Spike protein makes the SARS‐CoV‐2 more infectious than SARS, and TMPRSS2 (Transmembrane protease serine 2) is widely reported to mediate and assist in the invasion of host cells by multiple viruses. We found that these four genes were assigned to category C5, which had the corresponding HR label of “Spike protein (S) of coronavirus”. This demonstrated that TWIRLS can provide an interface to summarize human findings automatically and help human experts quickly understand the research directions and relevant knowledge in this field.

**FIGURE 3:**
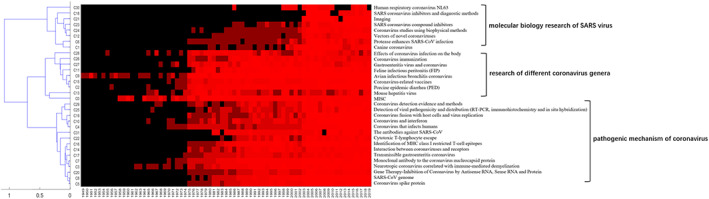
Cluster analysis of time information. Entity categories were grouped based on the distribution of date information corresponding to the topic entities contained in each category. In the heatmap, the rows represent the time information on a yearly basis and the columns represent the topic‐entity categories. To generate the heatmap/cluster dendrogram, Euclidean measure for distance matrix and complete agglomeration method for clustering was applied

At the same time, we also mined the time attribute of the entity from abstract text, and constructed the correlation matrix between the topic entities and years by counting the number of occurrences of each entity in the text sample in different years (1900–2019) (Table S5), and then clustered according to this time distribution information of each entity category. The result showed that the topic entity categories are divided into three groups based on the essence of the entity category labels: molecular biology research of SARS virus, research of different coronavirus genera and pathogenic mechanism of coronavirus. Further analysis of the time distribution information showed that the relevant literatures of the entity category in the molecular biology research group of SARS virus were mainly published after 2003, which is in line with the time point of SARS outbreak. Therefore, TWIRLS mines the research direction and trend of the topic categories from the time dimension (Figure 3).

The distribution and meaning of the data can be compared to specific expression values of CSHG under different conditions (here, the category is used as a condition). We applied general analysis method of pathway enrichment that the most relevant genes from each entity category are taken as input of the enrichment program for pathway analysis(Reimand et al., 2019). Therefore, TWIRLS can recommend the most likely and least likely signaling pathways based on the distribution of the pathway signatures (Table 2). On the other hand, TWIRLS can also recommend the most likely and least likely categories for each signaling pathway. As an example, Table 3 shows the signaling pathways most likely associated with category C3 and the most unlikely category.

**TABLE 2:** The most relevant and least relevant signaling pathways of each coronavirus‐entity category

Class	Likely pathway	Z score	Unlikely pathway	Z score
C0	PKCθ signaling in T lymphocytes	1.5782	Toll‐like receptor signaling	−1.7195
C1	AMPK signaling	5.1816	TGF‐β signaling	−4.0841
C2	Extrinsic prothrombin activation pathway	4.3314	Melanocyte development and pigmentation signaling	−3.8316
C3	Renin‐angiotensin signaling	5.6100	AMPK signaling	−4.4763
C4	April mediated signaling	3.2382	Neuregulin signaling	−2.4121
C5	Melanocyte development and pigmentation signaling	3.7887	Extrinsic prothrombin activation pathway	−3.9823
C6	Leukocyte extravasation signaling	2.3615	Pancreatic adenocarcinoma Siganling	−2.3694
C7	Role of BRCA1 in DNA damage response	4.6871	NF‐κB signaling	−4.8545
C8	PKCθ signaling in T lymphocytes	1.9902	VDR_RXR activation	−2.6143
C9	Toll‐like receptor signaling	4.2000	Role of BRCA1 in DNA damage response	−2.5017
C10	April mediated signaling	4.3113	TGF‐β signaling	−3.5853
C11	Acute phase response signaling	2.5066	Renin‐angiotensin signaling	−3.2492
C12	ATM signaling	2.7020	PKCθ signaling in T lymphocytes	−1.9846
C13	Retinoic acid mediated apoptosis signaling	2.6069	Interferon signaling	−1.8953
C14	ATM signaling	2.9244	PKCθ signaling in T lymphocytes	−1.9667
C15	AMPK signaling	1.9142	Leukocyte extravasation signaling	−2.4233
C16	Role of BRCA1 in DNA damage response	3.1033	Colorectal cancer metastasis signaling	−3.1033
C17	Production of nitric oxide and reactive oxygen species in macrophages	3.1398	mTOR signaling	−2.8893
C18	Extrinsic prothrombin activation pathway	1.8215	Intrinsic prothrombin activation pathway	−1.5680
C19	Role of NFAT in regulation of the immune response	2.0089	CD40 signaling	−2.2696
C20	April mediated signaling	2.3687	Aryl hydrocarbon receptor signaling	−1.5471
C21	NRF2‐mediated oxidative stress response	1.4679	CD40 signaling	−2.4042
C22	Toll‐like receptor signaling	2.2825	Melanocyte development and pigmentation signaling	−1.6007
C23	ATM signaling	2.5518	mTOR signaling	−1.8228
C24	Melanocyte development and pigmentation signaling	2.1700	Aryl hydrocarbon receptor signaling	−2.1700
C25	Extrinsic prothrombin activation pathway	1.9825	IL‐22 signaling	−1.7638
C26	mTOR signaling	2.7449	Aryl hydrocarbon receptor signaling	−1.7790
C27	AMPK signaling	2.4033	Intrinsic prothrombin activation pathway	−1.7579
C28	NRF2‐mediated oxidative stress response	2.0846	PKCθ signaling in T lymphocytes	−1.3415
C29	TGF‐β signaling	2.4379	Ephrin A signaling	−2.0030
C30	Role of NFAT in regulation of the immune response	2.1383	Leukocyte extravasation signaling	−1.8524
C31	Role of NFAT in regulation of the immune response	1.7979	Neuregulin signaling	−1.4467

**TABLE 3:** Recommended signaling pathway most relevant to entity category C3

Pathway	Likely class	Z score	Unlikely class	Z score
Renin‐angiotensin signaling	C3	5.6100	C5	−3.5918
VDR_RXR activation	C3	4.7060	C5	−3.2514
Aryl hydrocarbon receptor signaling	C3	4.3746	C5	−3.7887
Chemokine signaling	C3	3.9999	C7	−2.4881
IL‐8 signaling	C3	3.5211	C2	−2.6692
Neuregulin signaling	C3	3.2914	C1	−3.4134

### 3.3 Entity category‐associated genes involved in generalized interaction networks

**FIGURE 4:**
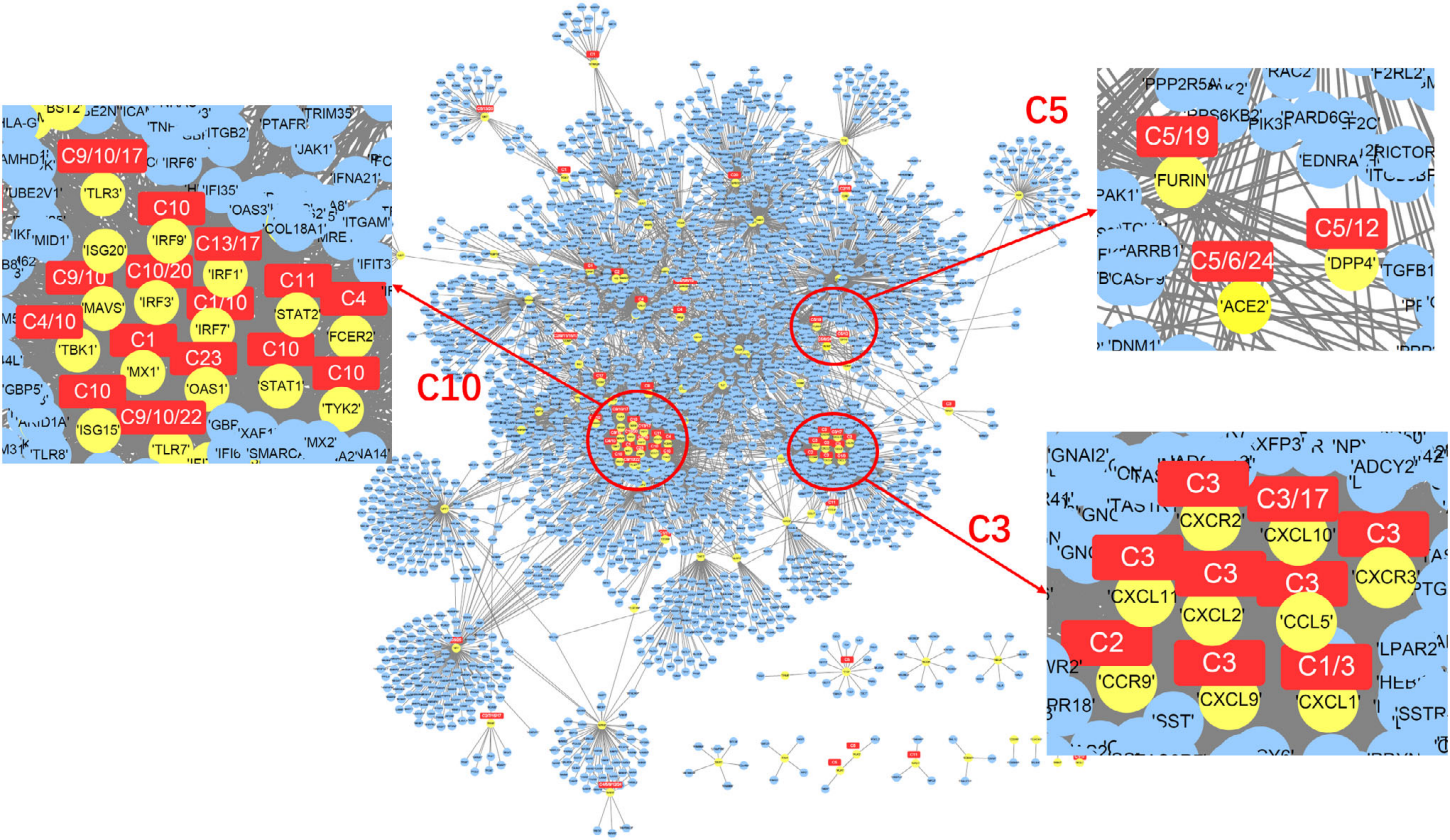
Gene interaction network centered on 119 CSHGs. The yellow nodes represent 119 CSHGs, the blue nodes represent genes that interact with CSHG in the string database (combination score > 800), and the red squares mark the most relevant entity category of CSHG

We coupled the above category information with gene interaction/regulation databases to construct a generalized protein–protein interaction network (PPI network) for 119 genes out of the 123 CSHGs. We defined the direct interaction between two genes as one degree (1°) of interaction, and the indirect interaction connecting two genes through a gene as two degrees (2°) of interaction. All the genes in the 1° networks mined in the PPI database are shown in Figure 4. The results after deduplication showed 2,004 pairs in the 119 CSHGs (see Table S6). As a control, the average interactions of 119 randomly selected genes in the database showed between 252 to 612 pairs (average 220.16, *SD* 35.15). Compared to random genes, the regulatory connections between CSHGs were significantly enriched (Z score = 50.97).

Those CSHGs associated with a certain category had much closer interactions. For example, CSHGs associated with category C3 (or associated with C5 or C10) were closer to each other in the 1° networks (Figure 4), suggesting that TWIRLS can possibly highlight important research directions and biology systems involved in coronavirus‐specific research and can provide reliable interfaces for further automatic inference.

Several hub genes among the 119 CSHGs were further recommended by TWIRLS. Compared with a random sampling from all interactions recorded in the database, these hub genes had significantly increased numbers of interactions with the other 118 CSHGs. The recommended results showed that the three members of the IFITMs family (IFITM1‐3) were ranked first, second, and sixth among the top 10 hub genes. The CSSE cloud of the IFITMs family genes is shown in Figure 2c–e and detailed ranking recommendation results are shown in Table S7. These IFITMs genes demonstrated 115 interactions, accounting for 8.59% out of all 1,338 interactions of the 119 CSHGs. These IFITMs were significantly enriched in the local samples representing updated coronavirus‐related studies (average 0.03% in the control test of random samplings, *p* < 1.5676e‐61). The IFITMs family plays crucial roles in the induction of interferons during viral infections. Under the action of interferon, IFITMs disrupt intracellular cholesterol homeostasis and prevent the virus from entering the host cell (Amini‐Bavil‐Olyaee et al., 2013). However, TWIRLS did not directly associate IFITMs with any category, and we will need further information so that TWIRLS can determine which part of these genes might be involved in the coronavirus infection and the host body response.

Combining the category information with generalized interaction databases provides richer interactions and regulatory linkages. We extended the 119 CSHGs to their 2° networks based on the interactions with higher likelihood connections (Combined score > 800). The 2° networks expanded the number of genes from 119 host genes to 3,494 genes that may be associated with coronaviruses (see Table S8 for a list of genes, excluding CS119, as this type of gene is called CSHG2). These genes are mainly involved in two types of functions: virus‐related signaling pathways and immune function‐related pathways. Table 4 shows a summary of the KEGG signaling pathways.

**TABLE 4:** The signaling pathways enriched by 119 CSHGs

KEGG_PATHWAY	Count	%	*p* value	Bonferroni	Benjamini	FDR
						
*Part 1. Virus related pathways*						
ptr05162:Measles	18	11%	1.05E‐13	1.38E‐11	1.38E‐11	1.22E‐10
ptr05164:Influenza A	19	11%	7.05E‐13	9.30E‐11	4.65E‐11	8.23E‐10
ptr05160:Hepatitis C	15	9%	2.30E‐10	3.04E‐08	7.60E‐09	2.69E‐07
ptr05161:Hepatitis B	11	7%	7.37E‐06	9.72E‐04	1.22E‐04	8.61E‐03
ptr05169:Epstein–Barr virus infection	6	4%	9.87E‐03	7.30E‐01	9.58E‐02	1.09E+01
ptr05168:Herpes simplex infection	14	8%	2.73E‐07	3.60E‐05	6.00E‐06	3.18E‐04
						
*Part 2. Immune functions*						
ptr04620:Toll‐like receptor signaling pathway	15	9%	6.78E‐12	8.95E‐10	2.98E‐10	7.92E‐09
ptr04062:Chemokine signaling pathway	16	10%	9.91E‐10	1.31E‐07	2.62E‐08	1.16E‐06
ptr04060:Cytokine‐cytokine receptor interaction	13	8%	2.80E‐06	3.69E‐04	5.28E‐05	3.27E‐03
ptr04622:RIG‐I‐like receptor signaling pathway	7	4%	1.41E‐04	1.84E‐02	2.06E‐03	1.64E‐01
ptr04623:Cytosolic DNA‐sensing pathway	6	4%	7.45E‐04	9.37E‐02	9.79E‐03	8.66E‐01
ptr04630:Jak–STAT signaling pathway	7	4%	6.11E‐03	5.54E‐01	7.09E‐02	6.90E+00
ptr04668:TNF signaling pathway	6	4%	7.61E‐03	6.35E‐01	8.06E‐02	8.53E+00

Among the entire network, we found several CSHGs in the 1° networks (32.6–35.71%) that directly interacted with three members of the IFITMs family, whereas fewer CSHGs in the 2° network (5.21–9.46%) indirectly interacted with them. Although there was a higher proportion of directly interacting CSHGs, they were not significantly enriched in any category (see Table S9 for the enrichment scores of the 1° network nodes in different categories), whereas the indirect CSHGs were significantly enriched mainly in the C3 and C10 categories (Z score > 3) (see in Table S10 for the enrichment scores of the 2° network nodes in different categories). These findings demonstrate that TWIRLS can provide new insights about hub molecules, particularly when coupled with interaction information. These new candidate IFITM genes had potential functions associated with category C3. However, after adding generalized interaction information, TWIRLS also inferred possible functions of these proteins not associated with any category.

### 3.4 Reconstruction of the mechanistic consequences of coronavirus invasion

Similar to the SARS virus, viral genomics and structural biology studies have shown that ACE2 is also a functional receptor for the new SARS‐CoV‐2 coronavirus, and the binding of ACE2 to the S protein in SARS‐CoV‐2 is 10 to 20 times stronger than in SARS (Wrapp et al., 2020), which may help the new coronavirus infect the host through the upper respiratory tract, significantly increasing its infectivity. Using TWIRLS, we were able to identify both ACE2 and DPP4 genes as CSHGs, and both were significantly associated with category C5. The HR label for this category is “associated with S protein.” Although entities in category C5 mainly show that virus invasion is facilitated by virus‐binding receptors and membrane proteases, the biological mechanism of receptor binding to viruses leading to pathological changes has been reported less frequently.

TWIRLS can also recommend new genes that interact with ^C5^CSHGs, and other 1° or 2° CSHGs linked to these genes might be enriched in other categories. These inferences are based on a process that finds new genes connected to different categories. The connected categories can suggest potential regulatory relationships between different biological functions or phenotypes. The genes that serve as linkers are potential targets for gain‐ and loss‐of‐function experiments to identify those systems described by the meaningful entities in these categories.

**FIGURE 5:**
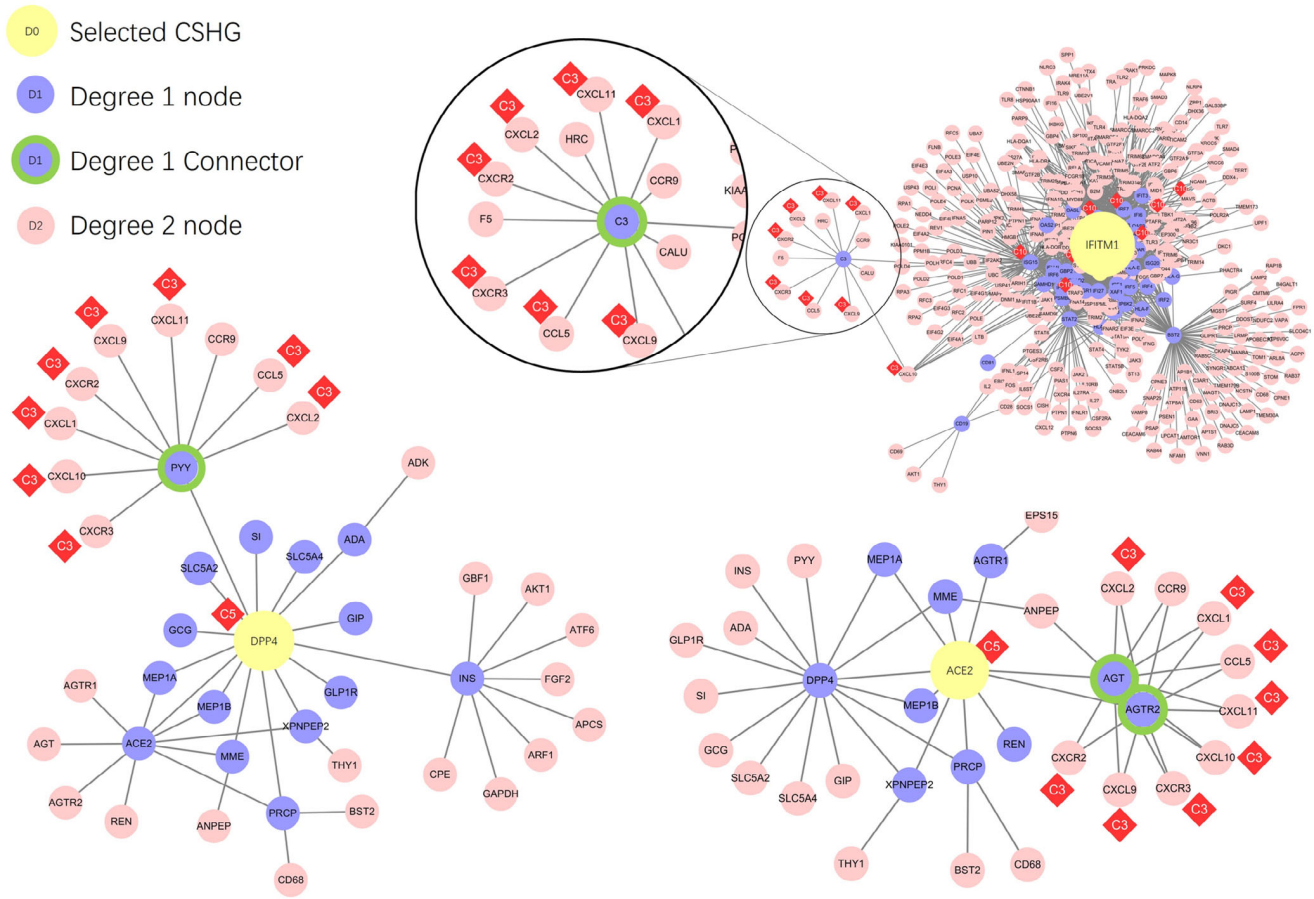
The gene interaction networks centered around DPP4, ACE2, and IFITM1, respectively. The yellow nodes represent the ACE2, DPP4 and IFITM1 genes, purple nodes represent genes that have 1° of interaction with the core genes, green circled purple nodes represent the genes connecting CSHG and C3 category‐related genes, and pink nodes represent genes with 2° of interaction with the core gene. The red diamonds show the most relevant entity category symbol for CSHG

In this study, TWIRLS found the 2° networks had connections with certain CSHGs associated with categories or with no category. For example, TWIRLS found that CSHGs in the 2° connections of IFITM1 were mainly concentrated in category C3 (see Figure 5). Interestingly, CSHGs in the 2° connections of ACE2 and DPP4 associated with category C5 were also enriched in category C3, inferring that the information summarized in category C3 probably describes the underlying mechanisms of the pathological changes after coronavirus infection. In our analysis, the signaling pathways in C3 were mainly RAS, Vitamin D and RXR activation, and Chemokine signaling, with RAS being the most significant (Table 3 shows a summary of the C3‐related signaling pathways).

Figure 5 shows that the CSHGs in the 2° connections of IFITM1, ACE2, and DPP4 were enriched in category C3 through different genes (AGT/AGTR2 in ACE2, PYY in DPP4, and C3 in IFITM1), which then linked to C3‐associated cytokines including CCL5, CXCL1, CXCL10, CXCL11, CXCL2, CXCL9, CXCR2, and CXCR3 (Figure 2f). Subsequently, these linker genes may contain information on the biological mechanisms that may be important for understanding these diseases. For example, TWIRLS recommended angiotensinogen (AGT) and angiotensin II receptor type 2 (AGTR2 or AT2R) genes in category C3 associated with ACE2. This supports that RAS is probably involved in the pathological changes caused by cytokine storms after S protein binds to ACE2 as suggested by other reports.

We next used TWIRLS to calculate the 1° and 2° networks of all 119 CSHGs. Based on the significantly enriched categories of CSHGs in the above networks, we used TWIRLS to construct separate models for the complex relationships of each CSHG. We found that 45.53% of the CSHGs in these networks were associated with C3 or C10 categories, and five genes (CCL3, CCL5, CXCL1, CXCL2, and STAT2) were associated with both categories. This suggests that the biological mechanisms described by C3 and C10 categories might be involved universally. Research on the entities, genes, pathways, and linker genes in the C3 and C10 categories could lead to new directions for the prevention, treatment, and clinical management of coronavirus infections.

### 3.5 Angiotensin II receptor blockers (ARBs) may be beneficial in patients with COVID‐19

**FIGURE 6:**
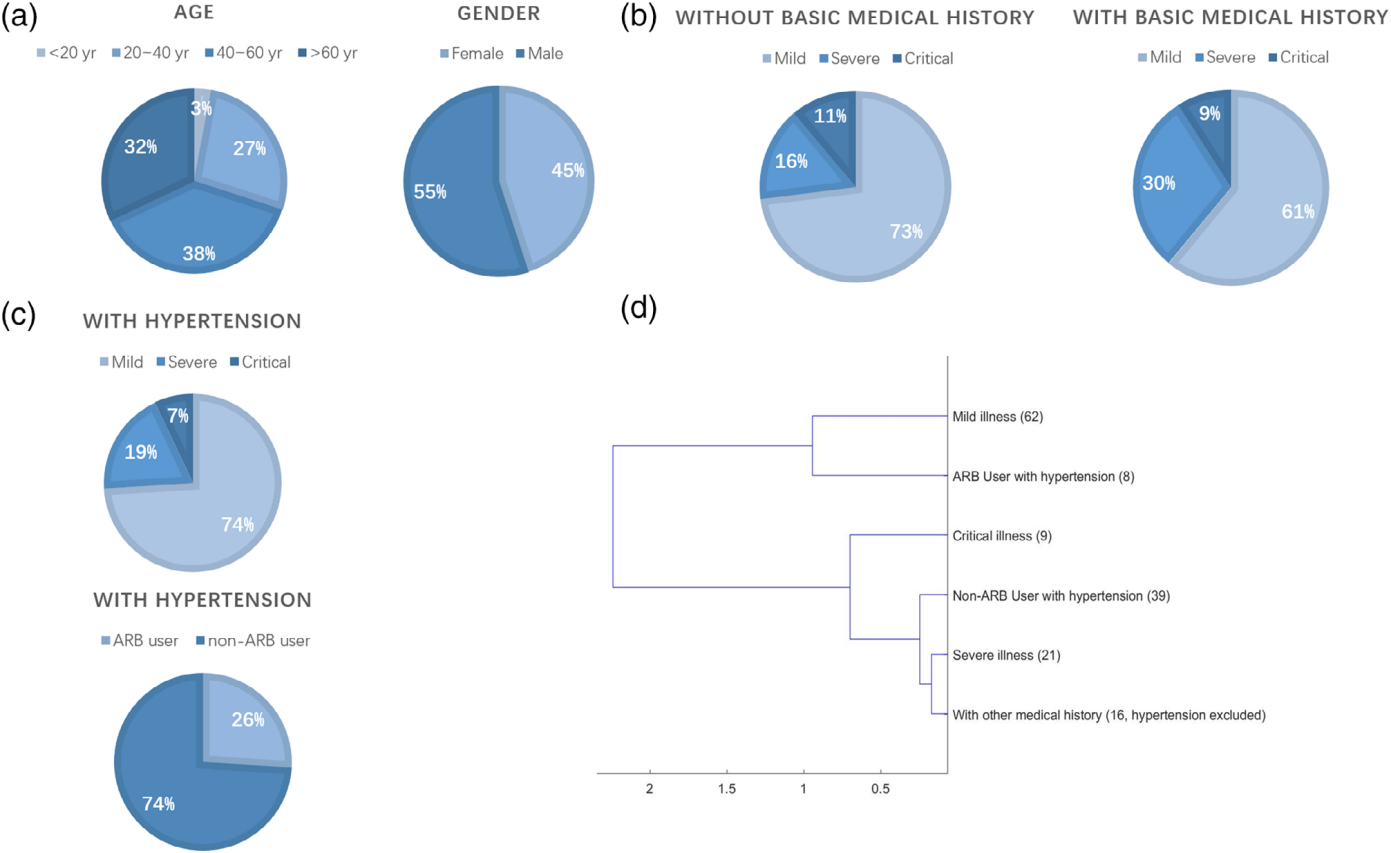
The specific distribution of COVID‐19 patient clinical data. (a) Age and gender distribution. (b) Distribution of mild, severe and critical illness in patients with or without basic medical history. (c) The distribution of illness and anti‐hypertension drug in patients with hypertension history. (d) Cluster graph of clinical characteristics of mild illness, severe illness, critical illness, ARB or non‐ ARB users with hypertension, and patients with other medical history

It has been demonstrated that the binding of the coronavirus spike proteins to ACE2 leads to ACE2 downregulation (Jia, 2016), which in turn results in unbalanced regulation of ACE‐Ang II axis and ACE2‐Ang‐(1–7) axis. The level of Ang‐(1–7) is then decreased, which affects angiogenesis inhibition, cardiovascular protection against oxidative stress, and significantly limits cell proliferation (Benter, Yousif, Anim, Cojocel, & Diz, 2006; Grobe, Mecca, Mao, & Katovich, 2006; Magaldi, Cesar, de Araújo, e Silva, & Santos, 2003; Shah, Oh, Lee, Lim, & Kim, 2012; Soto‐Pantoja, Menon, Gallagher, & Tallant, 2009). In contrast, the level of Ang II is relatively or absolutely elevated, which in turn contributes to lung injury, as angiotensin‐stimulated AT1R results in increased pulmonary vascular permeability, which mediates increased lung pathology(Imai et al., 2005; Kuba et al., 2005). Our results above also suggest that the homeostatic imbalance of RAS could be caused by viral binding to membrane ACE2 molecules, which may lead to dysregulation of inflammatory factor levels. Therefore, we evaluated the effects of the AT1R antagonists (ARB) such as losartan and telmisartan on the SARS‐CoV‐2 infection. We analyzed the medical records of 92 patients diagnosed with COVID‐19 pneumonia based on the New Coronavirus Pneumonia Prevention guidelines. More than one‐half of these patients (51.1%) had one or more underlying conditions including 31 patients (33.7%) with hypertension (Figure 6a–c).

Clinically, patients with COVID‐19 are classified into mild, severe, and critical according to the partial pressure of oxygen test, and in this study we can also define them by analyzing the differences in their blood indices. Using these blood indices we then investigated hypertension patients with and without ARB, and it turned out that they also can be clearly distinguished. Here 31 numeric blood indices in COVID‐19 patients were selected as the clinical characteristics, including the functional indices of the liver, kidney, and heart (Table S11). Patients with other medical history were also evaluated and clustered (see below).

For each index, we calculated the average of each group. As the numerical indices among various patients are not always normally distributed, we used a random distribution of the mean of the randomly separate group of patients for 10,000 times. According to the central limit theorem, the random distribution of any index should be normally distributed. Therefore, the Z score measures the statistical significance of each group of patient indexes defined by illness or treatments.

Patients with hypertension were divided into two groups, ARB users and non‐ARB users. Out of the 31 hypertensive patients, eight took ARB drugs (one took Telmisartan, two took Candesartan, three took Irbesartan and two took Valsartan) and the other 23 patients took other drugs such as calcium antagonists or diuretics before admission. After admission, all hypertensive patients were assigned a calcium antagonist for targeted treatment. We also considered 16 patients with other medical history without hypertension as the other patient group. For each group, the blood indices were compared between ARB and non‐ARB patients without any medical history to analyze differences with corresponding Z scores. We obtained Z scores of six groups representing the clinical characteristics of mild illness, severe illness, critical illness, ARB users, non‐ARB users, and patients without medical history, respectively (Table 5). The cluster analysis of the Z scores showed a closer relationship between non‐ARB users and severe illness (see Figure 6d), suggesting that ARB anti‐hypertensive drugs may have positive effects on reducing the severity of COVID‐19.

**TABLE 5:** The z score values of clinical characteristics

Clinical characteristics		ARB	Non‐ARB	With other medical history	Severe illness	Critical illness	Mild illness
Routine blood tests	White blood cells(WBC)count	1.35	1.06	0.90	1.93	−0.58	−0.32
	Neutrophil count(Neu)	1.42	1.47	1.23	2.77	0.03	−0.58
	Lymphocyte count(lymph)	−0.26	−1.37	−0.93	−2.78	−1.75	0.82
	Monocyte count(mono)	1.20	−0.21	−0.03	1.02	−1.63	0.02
	Neu%	1.18	2.51	1.43	3.49	1.52	−0.94
	Lymph%	−1.25	−2.58	−1.44	−3.66	−1.25	0.94
	Mono%	−0.27	−1.57	−0.76	−0.61	−0.85	0.24
	Red blood cell (RBC) count	0.76	−2.24	−1.05	−1.77	−0.48	0.43
	Hemoglobin(Hb)	0.55	−0.09	0.99	1.38	−0.04	−0.26
	Platelet(PLT)	0.41	0.97	0.72	0.97	−1.91	0.07
Liver function	Alanine aminotransferase (ALT)	−0.41	−0.73	−0.07	1.61	0.52	−0.40
	Aspartate aminotransferase (AST)	−0.68	0.39	0.58	0.22	2.99	−0.47
	Total bilirubin (TBIL)	1.01	0.92	0.11	0.34	0.20	−0.11
	Direct bilirubin (DBIL)	−0.04	−0.51	−0.13	−0.32	2.96	−0.37
	Indirect bilirubin (IBIL)	1.26	0.67	0.03	0.49	0.00	−0.11
	Gamma‐glutamyl transferase (GGT)	0.18	0.34	0.64	3.83	1.39	−0.97
	Alkaline phosphatase (ALP)	0.11	−0.01	−0.41	−0.30	−0.50	0.13
	Lactic dehydrogenase (LDH)	−0.54	0.32	0.74	2.70	3.55	−1.07
	Total protein (TP)	3.10	−0.73	−1.24	−1.11	−1.35	0.42
	Albumin (Alb)	1.97	−1.72	−2.22	−3.65	−1.52	0.99
	Globulin (Glb)	2.77	0.54	0.28	2.10	−0.47	−0.38
	ALB/GLB (A/G)	−1.20	−1.64	−1.54	−3.71	−0.49	0.86
Renal function	Na+	0.31	0.81	0.44	0.63	−3.49	0.37
	K+	1.31	−1.97	−0.47	−3.13	−1.36	0.83
	Fasting blood glucose (FBG)	0.03	1.94	0.05	0.41	−0.34	−0.05
	Uric acid(UA)	3.07	−1.99	−0.74	−0.84	−1.14	0.34
	Urea nitrogen(UN)	0.40	−0.55	−0.28	−0.21	−0.33	0.10
	Creatinine(Cr)	2.33	−1.57	−0.76	−0.51	−0.47	0.17
	Glomerular filtration rate(CDK‐EPI)	−2.83	−1.89	−0.43	−0.48	−1.34	0.29
Cardiac enzymes	Creatine kinase(CK)	−0.44	1.14	0.56	−0.26	2.79	−0.32
	Creatine kinase‐MB (CK‐MB)	−0.67	0.74	1.76	0.37	0.71	−0.18

### 3.6 Discussion

We used TWIRLS, a machine‐based approach, to collect, summarize, and analyze about 15,000 biomedical articles related to coronavirus, with the aim to elucidate the mechanisms underlying coronavirus‐induced host pathological changes. The TWIRLS system is an automated process that can be used to summarize the entities and genes related to coronavirus infection. By combining this system with generalized interaction databases, we can reveal further associations that can provide a deeper understanding of the biological mechanisms of the disease phenotype caused by virus‐host interactions. Using TWIRLS, we found a possible mechanism involving ACE2/AT2R‐RAS‐Cytokine signaling, which becomes imbalanced under virus infection leading to cytokine storms.

The Renin‐Angiotensin system consists of an enzymatic cascade beginning with liver‐mediated production of AGT (Skeggs, Dorer, Levine, Lentz, & Kahn, 1980). As part of RAS, angiotensin‐converting enzyme (ACE) regulates many physiological processes including inflammation and brain functions (Corvol, Eyries, & Soubrier, 2004). Angiotensin II (Ang II) is the main effector of this system and exerts most of its actions through the activation of Ang II type 1 and type 2 receptors (AT1R and AT2R)(Donoghue et al., 2000). Angiotensin II is formed by the successive enzymatic action of renin and ACE. Deficiency of ACE2 causes respiratory failure pathologies such as sepsis, pneumonia, and SARS (Boehm & Nabel, 2002; Imai et al., 2005). It has been confirmed that genetic deletion of AT1a receptor expression in mice can significantly improve lung function and reduce the formation of pulmonary edema compared with wild‐type mice (Sugaya et al., 1995). In contrast, inactivation of AT2R in mice aggravated acute lung injury. This suggests that AT1R mediates the pathogenicity of Ang II, whereas activated AT2R has a protective role(Hein, Barsh, Pratt, Dzau, & Kobilka, 1995). Thus, ACE/AT1R and ACE2/AT2R negatively feedback into one another, playing important roles in RAS‐mediated central nervous system and cardiovascular functions. The binding of the virus to ACE2 may disrupt this balance, which causes a homeostatic imbalance in RAS, leading to subsequent pathological changes.

**FIGURE 7:**
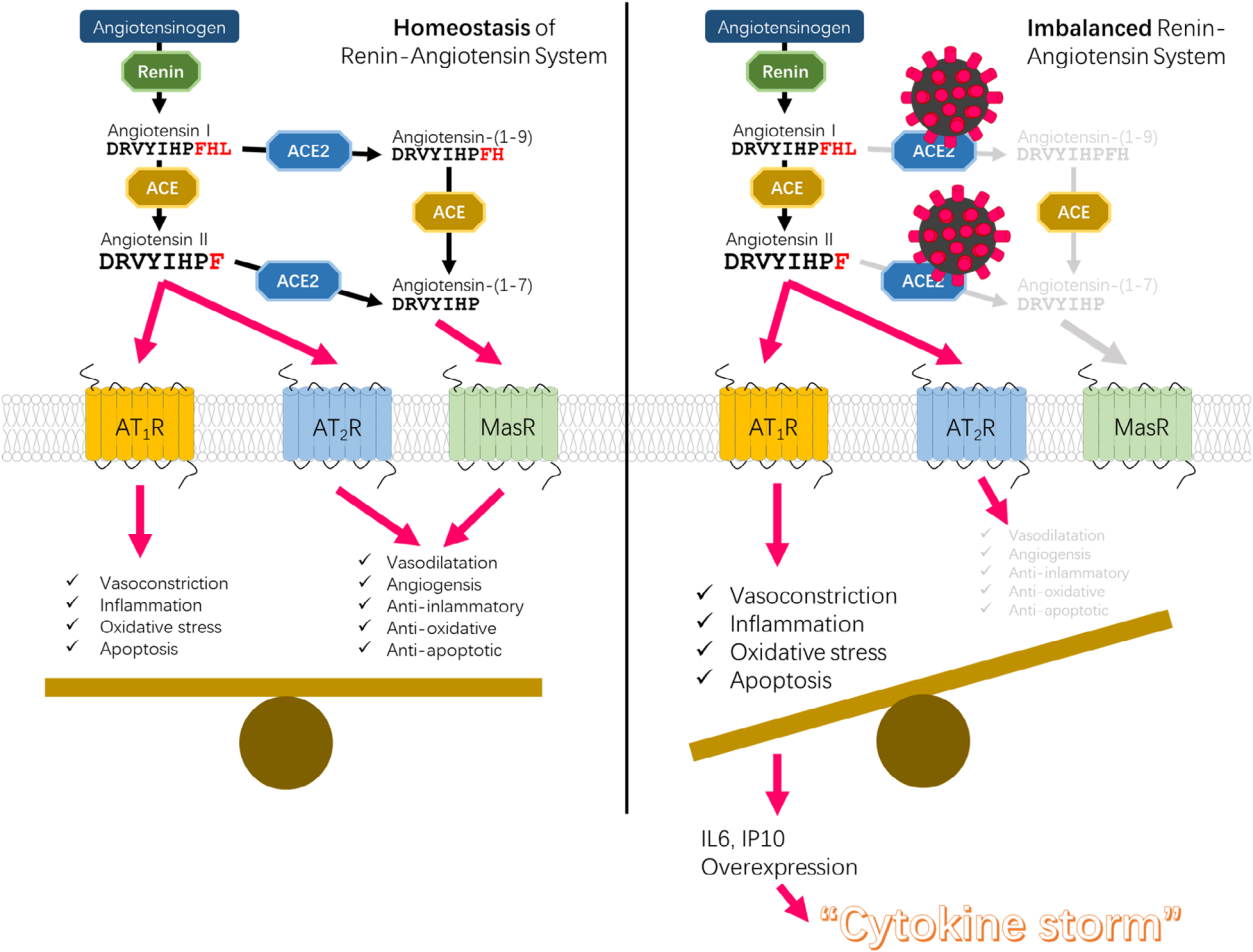
Disequilibrium of RAS‐cytokine signaling homeostasis causing cytokine storms triggered by ACE2‐ mediated coronaviral infection

Although Ang II was originally described as an effective vasoconstrictor, there is growing evidence that it is closely involved in the inflammatory response of the immune system. Proinflammatory cytokines derived from immune cells normally regulate the RAS component, which further accelerates the formation of systemic and local Ang II (Nataraj et al., 1999; Rudemiller & Crowley, 2016; Suzuki, Ruiz‐Ortega, Gomez‐Guerrero, Tomino, & Egido, 2003). In particular, proinflammatory cytokines regulate the production of AGT in the liver and kidney (Brasier, Ron, Tate, & Habener, 1990; Corvol & Jeunemaitre, 1997; Sriramula, Haque, Majid, & Francis, 2008). On the other hand, RAS has also been implicated in mediating the cytokine storm and has functional relationships with the immune system. Angiotensin II regulates vascular tension and stimulates the release of proinflammatory cytokines (El Bekay et al., 2003; Ruiz‐Ortega et al., 2002). The production and release of CXC chemokines can induce the accumulation of neutrophils in vivo (Ide, Hirase, Nishimoto‐Hazuku, Ikeda, & Node, 2008). Besides, ACE inhibitors and Ang II receptor blockers have been used in a number of cytokine‐mediated inflammatory pathologies, and AT1R blockers (angiotensin receptor blocker) were shown to have beneficial effects that are commonly attributed to AT2R activation (Henrion, 2012). It was also reported that Ang II‐stimulated human endothelial cells exhibit increased release of a CXC chemokine, IFN‐γ‐inducible protein 10 (IP‐10 or CXCL10). This protein is mainly expressed in the lung and is a chemoattractant for activated T cells. The expression of IP‐10 has been observed in many Th1‐type inflammatory diseases, where it is thought to play an important role in recruiting activated T cells to sites of tissue inflammation. Therefore, RAS dysfunction may result in the accumulation of cytokines in the lungs leading to excessive accumulation of immune cells and interstitial fluid, resulting in blocked airways and causing eventual death. In the first reports of severely infected patients diagnosed with COVID‐19, a large number of patients experienced “cytokine storms” that were fatal (Huang et al., 2020). Figure 7 summarizes the functional changes and pathological consequences of RAS after ACE2 combines with the coronavirus.

We expect the mechanism summarized and reasoned by TWIRLS can be further supported by pathological evidence. To date, only one report of a postmortem biopsy has been published with pathological data. Although histological examination showed bilateral diffuse alveolar damage with cellular fibromyxoid exudates, the right lung showed evidence of desquamation of pneumocytes and hyaline membrane formation, indicating acute respiratory distress syndrome (ARDS), whereas the left lung showed pulmonary edema with hyaline membrane formation, suggestive of early‐phase ARDS. The pathological evidence suggests that ARDS symptoms are closely related to cytokine storms (Xu et al., 2020). Based on the above results, we analyzed the clinical characteristics of COVID‐19 patients, which showed that patients taking ARBs were at a lower risk of developing severe lung damage than non‐ARB patients, indicating these anti‐hypertensive drugs may have positive effects on COVID‐19 patients.

Meanwhile, some latest hypothesizes also support this conclusion that angiotensin receptor 1 (AT1R) inhibitors might be beneficial for pneumonia patients infected by COVID‐19 (Gurwitz, 2020). In addition, the available evidence, in particular, data from human studies, does not support the hypothesis that using ACEI/ARB increases ACE2 expression and the risk of complications from COVID‐19 (Sriram & Insel, 2020). Therefore, we suggest that ARB can be used as potential alternatives for COVID‐19. At present, there are several ongoing clinical trials for testing the efficacy of ARB on COVID‐19 patients (telmisartan (Rothlin, Vetulli, Duarte, & Pelorosso, 2020), NCT04355936; losartan, NCT04312009; valsartan, NCT04335786). We hope that there will be more evidences of ARB clinical trials and more histopathology‐related data can further support our preliminary findings using machine approach. At the same time, in order to further rectify the deviation of structured knowledge generated by the algorithm, more rigorous data statistics methods, discussion and interviews with scientists are demanded for guaranteeing the goals of machine learning algorithm are consistent with that of the human. That it to say, only combining human experts and algorithms to realize machine learning with human guidance can really promote the development of machine learning in the future.

## DECLARATIONS

## CONFLICT OF INTEREST

The authors declare no competing interests.

## Supporting information


Supplementary Table S1

Supplementary Table S2

Supplementary Table S3

Supplementary Table S4

Supplementary Table S5

Supplementary Table S6

Supplementary Table S7

Supplementary Table S8

Supplementary Table S9

Supplementary Table S10

Supplementary Table S11

## Data Availability

All data generated or analyzed in this study are included in the published article. (Tables [Link], [Link], [Link], [Link]).
